# Pulsed ultrasound associated with gold nanoparticle gel reduces oxidative stress parameters and expression of pro-inflammatory molecules in an animal model of muscle injury

**DOI:** 10.1186/1477-3155-10-11

**Published:** 2012-03-12

**Authors:** Eduardo G Victor, Paulo CL Silveira, Jonathann C Possato, Guilherme L da Rosa, Uillian B Munari, Claudio T de Souza, Ricardo A Pinho, Luciano da Silva, Emilio L Streck, Marcos MS Paula

**Affiliations:** 1 Laboratory of Synthesis of Multifunctional Complexes, UNESC Av. Universitária, 1105 - Bairro Universitário Phone: + 55 48 34312773 88806-000 - Criciúma - SC, Brazil; 2 Laboratory of Physiology and Biochemistry of Exercise, UNESC Av. Universitária, 1105 - Bairro Universitário Phone: + 55 48 34312773 88806-000 - Criciúma - SC, Brazil; 3 Laboratory of Experimental Physiopathology, UNESC Av. Universitária, 1105 - Bairro Universitário Phone: + 55 48 34312773 88806-000 - Criciúma - SC, Brazil

**Keywords:** gold nanoparticles, oxidative stress, therapeutic pulsed ultrasound, anti-inflammatory, reactive oxidative species

## Abstract

**Background:**

Nanogold has been investigated in a wide variety of biomedical applications because of the anti-inflammatory properties. The purpose of this study was to evaluate the effects of TPU (Therapeutic Pulsed Ultrasound) with gold nanoparticles (GNP) on oxidative stress parameters and the expression of pro-inflammatory molecules after traumatic muscle injury.

**Materials and methods:**

Animals were divided in nine groups: sham (uninjured muscle); muscle injury without treatment; muscle injury + DMSO; muscle injury + GNP; muscle injury + DMSO + GNP; muscle injury + TPU; muscle injury + TPU + DMSO; muscle injury + TPU + GNP; muscle injury + TPU + DMSO + GNP. The ROS production was determined by concentration of superoxide anion, modulation of antioxidant defenses was determined by the activity of superoxide dismutase, catalase and glutathione peroxidase enzymes, oxidative damage determined by formation of thiobarbituric acid-reactive substance and protein carbonyls. The levels of interleukin-1β (IL-1β) and tumor necrosis factor-α (TNF-α) were measured as inflammatory parameters.

**Results:**

Compared to muscle injury without treatment group, the muscle injury + TPU + DMSO + GNP gel group promoted a significant decrease in superoxide anion production and lipid peroxidation levels (p < 0.050). It also showed a significant decrease in TNF-α and IL-1β levels (p < 0.050) when compared to muscle injury without treatment group.

**Conclusions:**

Our results suggest that TPU + DMSO + GNP gel presents beneficial effects on the muscular healing process, inducing a reduction in the production of ROS and also the expression of pro-inflammatory molecules.

## Background

Muscle contusion usually results from a direct blunt impact and is frequently associated with contact sports. Muscle contusion results from microscopic muscle fiber and capillary disruption with resultant microhemorrhage dissecting between the torn fibers and the remaining viable muscle fibers [1]. Muscle injury typically initiates a rapid and sequential invasion of muscle by inflammatory cell populations that can persist for days to weeks, while muscle repair, regeneration, and growth occur [2].

Many studies have demonstrated that muscle injury induces an increased generation of Reactive Oxidative Species (ROS), which alters intracellular oxidant-antioxidant balance in favour of the former and can result in oxidative damage of traumatized muscle when the production of ROS overwhelms the antioxidant defense systems [3]. Oxidative stress has an important role in muscle damage and may be associated with cellular membrane damage via disruption of Ca^2+ ^ion channels leading to imbalance in Ca^2+ ^homeostasis [4]. Multiple potential sites for ROS generation in skeletal muscle have been identified including mitochondria, NADPH oxidase enzymes, phospholipase A2-dependent processes, and xanthine oxidase [5].

The cytokines further promote the migration, proliferation and survival of various cell types at the injury site, whereas the inflammatory cells are responsible for the phagocytosis of cell debris [6]. Macrophage-released cytokines, including leukemia inhibitory factor, IL-1 and IL-6, have emerged as part of the muscle repair mechanism. In addition, it is increasingly clear that tumor necrosis factor-α (TNF-α), a major pro-inflammatory cytokine produced by activated macrophages, has an important role in muscle repair [7].

Recent studies concluded that some physical methods including therapeutic ultrasound treatments accelerate and facilitate wound healing, improve scar quality, and have beneficial effects on muscle and tendon healing [8]. Therapeutic ultrasound is used to promote healing in a variety of situations, such as in reducing edema, accelerating tissue repair, decreasing pain, and modifying the formation of scar tissue. Furthermore, ultrasound may enhance penetration of anti-inflammatory agents applied to the skin in a technique called phonophoresis [9]. Phonophoresis has been used clinically as a physical therapy for musculoskeletal lesions. Therefore, anti-inflammatory drugs and local anesthetics have been frequently administered via this route [10].

Nanogold (also called gold nanoparticle or colloidal gold) has been actively investigated in a wide variety of biomedical applications due to its biocompatibility and ease of conjugation to biomolecules [11,12]. According to Tsai (2007) [13], nanogold has anti-inflammatory effect by decreasing pro-inflammatory cytokines and macrophage infiltration in a model of arthritis.

Due to anti-inflammatory properties of therapeutic pulsed ultrasound (TPU) and the growing interest in the research of gold nanoparticles, the present study aims at linking these two treatments (phonophoresis), evaluating their effects on the cytokines TNF-α and IL-1β and oxidative stress parameters after traumatic muscle injury.

## Materials and methods

### Synthesis of Gold Nanoparticles

Gold nanoparticles were prepared as previously described by Turkevich et al. (1951) [14]. Briefly, an aqueous solution of sodium citrate was added to a hydrogen tetrachloroaurate (HAuCl_4_) solution previously heated to 90°C. The system was maintained under reflux with magnetic stirring for 20 min. Gold nanoparticles were characterized by UV-vis and TEM (transmission electron microscopy). The electron spectrum shows an absorption peak at approximately 520 nm, attributed to the surface plasmon resonance absorption. TEM image of nanoparticles revealed the presence of nearly spherical particles with mean particles diameter of 25 nm.

### Animals

Male Wistar rats (250-300 g) obtained from the Central Animal House of the Universidade do Extremo Sul Catarinense (UNESC), Santa Catarina, Brazil, were caged in groups of six, offered commercial rat chow and water *ad libitum*, and maintained on a 12 h light/12 h dark cycle. The animals were randomly divided into nine groups (n = 6): sham (uninjured muscle); muscle injury without treatment; muscle injury and treatment with DMSO gel (15 mg/kg); muscle injury and treatment with GNP gel (27 μg); muscle injury and treatment with DMSO + GNP gel; muscle injury and TPU (0.8 W/cm^2^) + saline gel (0.9%); muscle injury and TPU (0.8 W/cm^2^) + DMSO gel (15 mg/kg); muscle injury and TPU (0.8 W/cm^2^) + GNP gel (27 μg); muscle injury and TPU (0.8 W/cm^2^) + DMSO + GNP gel. All studies were performed in accordance with guidelines of National Institutes of Health and with the approval of the Ethics Committee of the Universidade do Extremo Sul Catarinense, Santa Catarina, Brazil.

### Muscle injury model

The muscle trauma model used has been described by Rizzi et al. (2007) [15]. Animals were anesthetized with intraperitoneal injection of ketamine (70 mg/kg) and xylazine (15 mg/kg). Gastrocnemius injury was induced by a single-impact blunt trauma in a press developed by the Centro Industrial de Equipamentos de Ensino e Pesquisa (CIDEP, Porto Alegre, RS, Brazil). Briefly, injury was produced by a metal mass (0.459 kg) falling through a metal guide from a height of 18 cm. The impact kinetic energy delivered was 0.811 Joules. Sham rats were also anesthetized to ensure standardization, but without muscle trauma.

### Treatment

Treatment with TPU(Imbramed, Amparo, São Paulo, Brazil, 6 min duration, frequency of 1.0 MHz, intensity of 0.8 W/cm^2^, effective radiating area [ERA] 1 cm^2^, 50% duty cycle of 1:2 [5 ms on, 5 ms off] and focused geometry of the ultrasound beam) was used 2, 12, 24, 48, 72, 96, 120, 144, and 168 h after muscle trauma, adapted from Silveira et al. (2010) [16]. The ultrasound treated area had approximately 2 cm^2^, according to Rizzi et al. (2007) [15]. The movement of the beam was circular, according to Saliba et al. (2007) [17]. The groups with muscle injury and treatment with DMSO gel, GNP gel, and DMSO + GNP gel also were exposed to treatment for 6 min.

### Sacrifice protocol

Two hours after the last application, animals were killed by decapitation. The injured region of the gastrocnemius muscle was surgically removed and immediately processed, aliquoted and stored at -70°C for subsequent analysis.

### Sample preparation

The injured region of the gastrocnemius was homogenized in the buffer used for each technique. The homogenates were centrifuged at 1000 g for 10 min at 4°C and the supernatants kept at -70°C until used in the experiments. The maximal period between homogenate preparation and biochemical analysis was always less than 5 days.

### Biochemical Assays

#### Measurement of mitochondrial superoxide generation

Submitochondrial particles were isolated by differential centrifugation, as previously described by Poderoso et al. (1996) [18]. Superoxide anion production was estimated by measuring adrenaline oxidation in a buffer containing submitochondrial particles, succinate (as electron transfer chain initiator) and catalase. Results were expressed in nmol/min/mg protein.

#### Lipid peroxidation

The formation of thiobarbituric acid-reactive substances (TBARS) during a TBA-heating reaction was used as an index of lipid peroxidation, as previously described by Draper and Hadley (1990) [19]. Briefly, the samples were mixed with 1 mL of 10% trichloroacetic acid and 1 mL of 0.67% thiobarbituric acid. Subsequently, they were heated in a boiling water bath for 30 min. TBARS level was determined through absorbance at 532 nm using 1,1,3,3-tetramethoxypropane as an external standard. Results were expressed as TBARS level (nmol/mg protein).

#### Protein carbonyls

Oxidative damage to proteins was measured by determining carbonyl groups based on the reaction with dinitrophenylhydrazine (DNPH) [20]. Proteins were precipitated by adding 20% trichloroacetic acid and reacted with DNPH. The samples were then redissolved in 6 M guanidine hydrochloride and carbonyl contents were determined through absorbance at 370 nm using a molar absorption coefficient of 22.000 M^-1 ^and total protein were determined by absorbance of 270 nm in the same sample. Results were expressed as carbonyl level (nmol/mg protein).

#### Superoxide dismutase (SOD) activity

SOD activity was assayed by measuring the inhibition of adrenaline auto-oxidation as absorbance at 480 nm, as previously described Bannister and Calabrese (1987) [21]. Results were expressed in U SOD/mg protein.

#### Glutathione peroxidase (GPx) assay

GPx activity was measured by using tert-butyl-hydroperoxide as substrate [22]. Enzyme activity was measured by monitoring the rate of disappearance of NADPH at 340 nm in 50 mM potassium phosphate buffer, pH 7.0, containing 1.0 mM EDTA, 2.0 mM glutathione (GSH), 0.2 U/mL GSH reductase, 1.0 mM azide, 0.2 mM tert-butyl-hydroperoxide, 0.2 mM NADPH, and supernatant containing 0.2-0.3 mg protein/mL.

GPx activity was expressed as nmol of NADPH oxidized per minute per mg of protein, using an extinction coefficient of 6.22 × 10^6 ^for NADPH.

#### Catalase (CAT) activity

Catalase activity was measured by the rate of decrease in hydrogen peroxide absorbance at λ_max _= 240 nm [23], and results were expressed in U CAT/mg protein.

#### Protein analysis by immunoblotting

The injured region of the gastrocnemius muscle was surgically removed and homogenized immediately in extraction buffer (1% Triton-X 100, 100 mM Tris, pH 7.4, containing 100 mM sodium pyrophosphate, 100 mM sodium fluoride, 10 mM EDTA, 10 mM sodium vanadate, 2 mM PMSF, and 0.1 mg of aprotinin/mL) at 4°C with a Polytron MR 2100 (Kinematica, Switzerland). The extracts were centrifuged at 11000 rpm and 4°C in an Eppendorf 5804R (Eppendorf AG, Hamburg, Germany) for 40 min to remove insoluble material, and the supernatants of this tissue were used for protein quantification, according to the Bradford method [24]. Proteins were denaturated by boiling in Laemmli [25] sample buffer containing 100 mM DTT, run on SDS-PAGE, transferred to nitrocellulose membranes. Membranes were blocked, probed, and developed. Antibodies used for immunoblotting were anti-TNF-α, anti-IL-1β (Santa Cruz Biotechnology, Santa Cruz, CA, USA). Chemiluminescence detection was performed with horseradish peroxidase-conjugate secondary antibodies. Autoradiographs of membranes were taken for visualization of protein bands. The results of blots are presented as direct comparisons of bands in autoradiographs and quantified by densitometry using the Scion Image software.

#### Protein determination

The amount of protein in the samples tested for TBARS, protein carbonyl and enzymes activities were determined using the Lowry technique [26].

### Statistical analysis

Data were analyzed by one-way analysis of variance (ANOVA) followed by Tukey's test when p-values were significant (p < 0.050). All analyses were performed using the Statistical Package for the Social Science (SPSS, v 17.0; IBM Corp, Armonk, NY) software.

## Results

### Superoxide anion production

**Figure 1:**
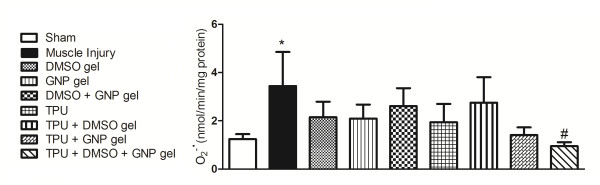
**Effect of therapeutic pulsed ultrasound (TPU) + DMSO + GNP gel on anion superoxide production in skeletal muscle after injury (seven days)**. Data are expressed as mean ± SEM for six animals. Different from sham (*p < 0.050) and different from muscle injury without treatment (**^#^**p < 0.050). (Tukey's test).

The group of rats with muscle injury and no treatment had a significant increase in relation to the sham group (p = 0.043), and only the TPU + DMSO + GNP gel group showed a significant decrease compared to the muscle injured without treatment group (p = 0.023) (Figure 1).

### Lipid peroxidation and protein carbonyls

**Figure 2:**
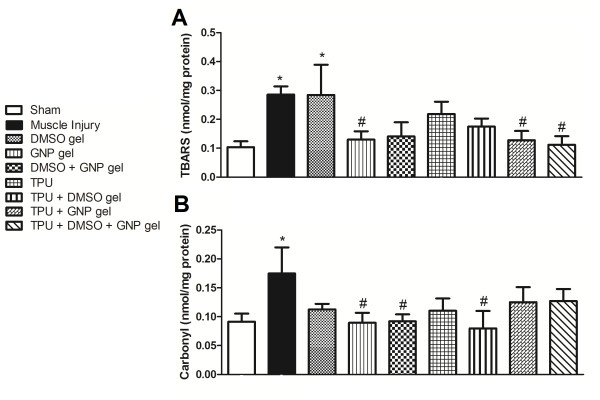
**Effect of therapeutic pulsed ultrasound (TPU) + DMSO + GNP gel on thiobarbituric acid reactive substances (TBARS) **(A) **and protein carbonyl **(B) **levels in skeletal muscle after injury (seven days)**. Data are expressed as mean ± SEM for six animals. Different from sham (*p < 0.050) and different from muscle injury without treatment (^#^p < 0.050). (Tukey's test).

The levels of lipid peroxidation of the groups of rats with muscle injury and no treatment group (p = 0.007) and the DMSO gel group (p = 0.007) presented a significant increase compared to the sham group. Similarly, the GNP gel (p = 0.025), TPU + GNP gel (p = 0.023), and TPU + DMSO + GNP gel (p = 0.013) groups presented a significant decrease when compared to the muscle injury group without treatment (Figure 2A). Protein carbonylation was also significantly higher in the muscle injury group without treatment when compared to the sham group (p = 0.017). The GNP gel (p = 0.020), DMSO + GNP gel (p = 0.035), and TPU + DMSO gel (p = 0.010) groups showed a significant decrease compared to the muscle injury group without treatment (Figure 2B).

### Superoxide dismutase, glutathione peroxidase and catalase activities

**Figure 3:**
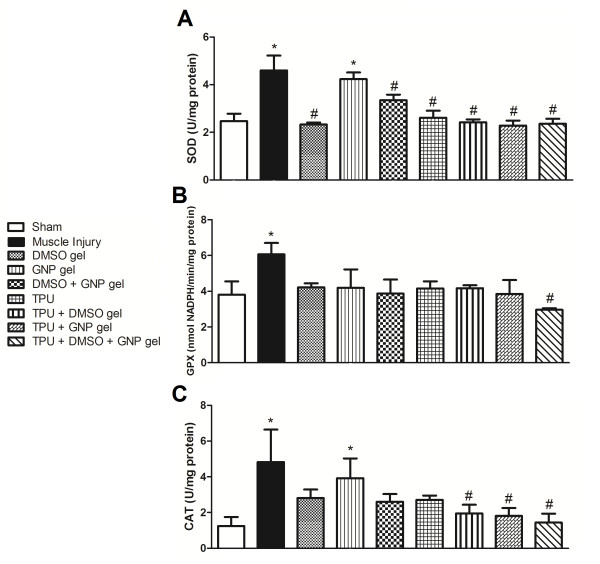
**Effect of therapeutic pulsed ultrasound (TPU) + DMSO + GNP gel on superoxide dismutase **(A)**, glutathione peroxidase **(B) **and catalase **(C) **levels in skeletal muscle after injury (seven days)**. Data are expressed as mean ± SEM for six animals. Different from sham (*p < 0.050) and different from muscle injury without treatment (^#^p < 0.050). (Tukey's test).

When analyzing superoxide dismutase activity, the muscle injury without treatment (p = 0.001) and GNP gel (p = 0.006) groups presented a significant increase in relation to the sham group. The DMSO gel (p = 0.001), DMSO + GNP gel (p = 0.013), TPU (p = 0.003), TPU + DMSO gel (p = 0.001), TPU + GNP gel (p = 0.001), and TPU + DMSO + GNP gel (p = 0.001) groups showed a significant decrease in relation to the muscle injury without treatment (Figure 3A).

With regard to glutathione peroxidase activity, the muscle injury without treatment group (p = 0.035) had a significant increase in relation to the sham group, and only the TPU + DMSO + GNP gel (p = 0.021) group showed a significant decrease in relation to the muscle injury without treatment group (Figure 3B).

As for catalase activity, the muscle injury without treatment (p = 0.002) and GNP gel (p = 0.017) groups showed a significant increase in relation to the sham group. Similarly, the TPU + DMSO gel (p = 0.008), TPU + GNP gel (p = 0.008), and TPU + DMSO + GNP gel (p = 0.002) groups showed a significant decrease in relation to the muscle injury without treatment (Figure 3C).

### Pro-inflammatory Cytokines

**Figure 4:**
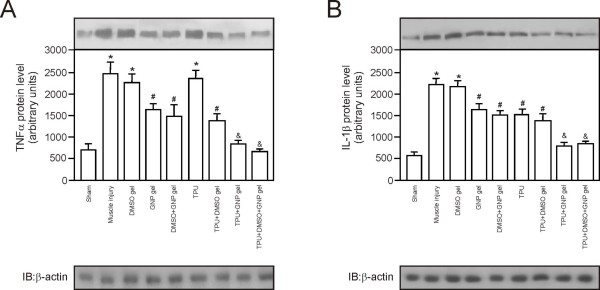
**Effect of therapeutic pulsed ultrasound (TPU) + DMSO + GNP gel on protein levels of cytokines in skeletal muscle after injury (seven days)**. Protein levels of TNF-α **(A) **and IL-1β **(B)**. *p < 0.050 *versus *Sham group; ^#^p < 0.050 *versus *muscle injury group; and ^&^p < 0.050 *versus *all other groups except sham.

With regard to TNF-α levels, the muscle injury without treatment (p = 0.000) DMSO gel (p = 0.001) and TPU (P = 0.000) groups had a significant increase in relation to the sham group. The GNP gel (p = 0.040), DMSO + GNP gel (p = 0.035), TPU + DMSO gel (p = 0.035) groups presented a significant decrease in the levels of TNF-α in relation to the muscle injury without treatment group. The TPU + GNP gel and TPU + DMSO + GNP gel groups showed a significant decrease compared to all other groups except sham (Figure 4A).

We also evaluate IL-1β levels and here the muscle injury without treatment (p = 0.000) and DMSO gel (p = 0.000) groups showed a significant increase compared to the sham group. Similarly, the GNP gel (p = 0.025), DMSO + GNP gel (p = 0.016), TPU (p = 0.020), and TPU + DMSO gel (p = 0.011) groups showed a significant decrease compared to the muscle injury without treatment group. On the other hand, the TPU + GNP gel and TPU + DMSO + GNP gel groups showed a significant decrease compared to all other groups except sham (Figure 4B).

## Discussion

Studies conducted over the past 15 years show that reactive oxidative species (superoxide, hydroxyl radicals, nitric oxide, peroxynitrite, and the free radical-derived product hydrogen peroxide) play an important role in inflammation and or infection-induced alterations in muscle function [27]. In the present study, we induced a traumatic muscle injury and assessed oxidative stress parameters after seven days. Figures 1, 2 and 3 show that there was an increase in superoxide anion production, TBARS levels, and carbonyl content, as well as in the activity of the antioxidant enzymes SOD, GPX and CAT in the muscle lesion group when compared to the sham.

After initial muscle injury, oxidative stress might have increased due to a number of potential sites for the generation of ROS within the traumatized muscle [28]. Primary sources of free radicals may include mitochondria, xanthine oxidase enzymes (XO), prostanoid metabolism, and NAD(P)H oxidases [29]. Reactive oxidative species are potent oxidizing and reducing agents which cause cell membrane damage by lipid peroxidation and also induce damage to proteins by neutrophil activation [30]. Cell injury activates cyclooxygenase and lipoxygenase pathways along with transition metal ions, which increase lipid peroxidation and protein carbonylation in the surrounding tissues [31].

However, in our treatment groups, only the TPU + DMSO + GNP gel group showed a significant decrease in superoxide anion production and TBARS levels, and it was the only group to show a decrease in all antioxidant enzyme levels compared to the muscle injury group.

TPU by transmitting as an acoustic pressure wave and indirectly applying mechanical stress to the tissues has been reported to promote protein synthesis, calcium uptake, and DNA synthesis in different cells [32-34]. Furthermore, ultrasound may enhance penetration of anti-inflammatory agents applied onto the skin in a technique called phonophoresis due to the cavitation phenomenon, the generation of gas bubbles, which oscillate and may implode at the skin surface, thus producing disorganization and/or an aqueous pathway through the *stratum corneum *[35-37]. Taking advantage of these properties, we introduced gold nanoparticles in the conductor gel of the pulsed ultrasound, as Figures 1, 2, and 3 show, antioxidant effects were observed.

Gold preparations used in Indian medicine systems (Ayurveda, Siddha and Unani-Tibb), have been attributed various medicinal uses. The metal is widely used in modern medicine for the treatment of rheumatoid arthritis [38]. Recent studies [39-42] demonstrated that metal nanoparticles are potential antioxidants. Bimetallic nanoparticles consisting of gold and platinum are effective in quenching reactive oxidative species (ROS), including hydrogen peroxide (H_2_O_2_) and the superoxide anion radical (O_2_^-•^), in dose-dependent manner and gold nanoparticles enhance the antioxidant activity of vitamin E. In addition, gold and platinum nanoparticles have been found to catalyze oxidation of NADH to NAD.

In general, we believe that the effects on oxidative stress of both therapies are improved when used together. To better understand this mechanism, we have also evaluated the effects of phonophoresis with gold nanoparticles on TNF-α and IL-1β levels.

In our study, we show that the muscle lesion model increases TNF-α and IL-1β levels compared to the sham group. After muscle injury, there is a fast necrosis of the muscle fibers and activation of inflammation, which contributes to the removal of necrotic material and secretion of several cytokines and growth factors, stimulating satellite cell activation [43]. Immediately after an injury to skeletal muscle, the gap formed between the ruptured muscle fibers is filled with a hematoma, where macrophages and fibroblasts are activated, producing additional chemotactic signals (e.g. growth factors, cytokines, and chemokines) for the circulating inflammatory cells [44].

However, the DMSO gel group showed a significant increase in TNF-α and IL-1β levels compared to the muscle injury group, while the TPU + GNP gel and TPU + DMSO + GNP gel group showed a significant decrease compared to all groups except sham.

During the inflammatory phase of the healing process, ultrasound can activate immune cells to migrate to the lesion site. In two separate studies, Fyfe et al. (1982, 1984) showed ultrasonic induction (0.5 W/cm^2^) of mast cell degranulation and histamine release in injury models *in vivo *[45,46]. Similar results were reported for dermal mast cells, demonstrating that ultrasound can accelerate the inflammatory healing phase for skin lesions/ulcers *in vivo *(Wistar rats, 0.75-3 MHz, 0.25-3 W/cm^2^) [45,46].

TPU stimulate a better response to the inflammatory process; however, the TPU + GNP gel and TPU + DMSO + GNP groups showed the highest decrease in TNF-α and IL-1β levels. Ultrasound is known to facilitate molecule transit across membranes. Ultrasound stimulation may influence the activity of plasma membrane Ca^2+^-ATPase, H^+^-ATPase, and other ion channels [47]. Due to these properties, TPU can be used together with anti-inflammatory or antioxidant drugs (phonophoresis) promoting a higher absorption and potentiating their effects [48,49].

Gold compounds have received great attention as anti-inflammatory agents due to their ability to inhibit expression of NF-κB and subsequent inflammatory reactions [50,51]. Auranofin (AF; 2,3,4,6-tetra-O-acetyl-1-thio-(-D-glucopyranosato-S-[triethylphosphine]gold) is a sulfur-containing gold compound, which has been widely used for the treatment of rheumatoid arthritis [52]. It has been shown that AF has anti-inflammatory and immunosuppressive activities. The drug blocks NF-κB activation by interacting with *cys*-179 of IKK-β and inhibits the production of pro-inflammatory cytokines such as IL-1β and TNF-α [53]. In fact, we believe that the association between TPU and GNP promotes major changes in muscle inflammation.

## Conclusions

Our findings suggest that phonophoresis with gold nanoparticles has antioxidant effect and, consequently, it decreases pro-inflammatory cytokine levels. Our results show that this treatment decreases the injured tissues exposure to reactive oxidative species, thus decreasing structural damages caused by this exposure and probably hastening the acute inflammatory phase.

Thus, gold nanoparticles suggest a promising application in the recovery of muscle lesions; however, further studies are needed in order to elucidate their precise mechanism of action.

## Competing interests

The authors declare that they have no competing interests.

## Authors' contributions

EGV, PCLS, JCP, GLR and UBM performed all necessary experiments, EGV, PCLS, CTS, RAP, LS, ES and MMSP analyzed data and wrote manuscript. All authors read and approved the final manuscript.
